# Intestinal tuberculosis in a child living in a country with a low incidence of tuberculosis: a case report

**DOI:** 10.1186/1756-0500-7-762

**Published:** 2014-10-27

**Authors:** Piera Dones, Maria Di Gangi, Maria Concetta Failla, Selene Genova, Caterina Giannitto, Giovanni Corsello, Nicola Principi, Susanna Esposito

**Affiliations:** Pediatric Infectious Disease Unit, Di Cristina Hospital, Palermo, Italy; Radiology Unit, Fondazione IRCCS Ca’ Granda Ospedale Maggiore Policlinico, Milan, Italy; Department of Neonatology and Pediatrics, University of Palermo, Di Cristina Hospital, Palermo, Italy; Pediatric Highly Intensive Care Unit, Department of Pathophysiology and Transplantation, University of Milan, Fondazione IRCCS Ca’ Granda Ospedale Maggiore Policlinico, Via Commenda 9, 20122 Milano, Italy

**Keywords:** Emerging infections, Gastrointestinal infections, Intestinal tuberculosis, *Mycobacterium tuberculosis*, Tuberculosis

## Abstract

**Background:**

Relatively common in adults, intestinal tuberculosis is considered rare in children and adolescents. The protean manifestations of intestinal tuberculosis mean that the diagnosis is often delayed (sometimes even for years), thus leading to increased mortality and unnecessary surgery. The main diagnostic dilemma is to differentiate intestinal tuberculosis and Crohn’s disease because a misdiagnosis can have dramatic consequences.

**Case presentation:**

A 13-year-old Caucasian, Italian female adolescent attended the Emergency Department complaining of abdominal pain, a fever of up to 38°C, night sweats, diarrhea with blood in stool, and a weight loss of about three kilograms over the previous two months. Physical examination revealed a marked skin pallor and considerable abdominal distension with relevant discomfort in all the abdominal quadrant. Laboratory tests revealed a decreased white blood cell count with anemia and increased C-reactive protein levels. The Mantoux tuberculin skin test was negative. A chest X-ray and an abdominal ultrasonography did not reveal any significant findings. The patient underwent colonoscopy that showed diffuse mucosal congestion and significant blood loss, and laparatomy showed small bowel and colon loops with a whitish appearance. A biopsy of the ileal mucosa revealed inflammation with noncaseating granulomas possibly due to bacterial infection. Given the suspicion of an opportunistic bacterial infection in a child with chronic inflammatory bowel disease (possibly Crohn’s disease), treatment with a third-generation cephalosporin was started. However, the abdominal pain, fever and poor general condition persisted and so, after 11 days, the patient underwent total body computed tomography and magnetic resonance imaging of the brain. On the basis of the radiological findings, miliary tuberculosis was suspected and bronchoscopy was performed and resulted positive for *Mycobacterium tuberculosis*. Miliary tuberculosis was confirmed and an effective treatment with four drugs was started.

**Conclusion:**

This case shows that the manifestations of intestinal tuberculosis can be very difficult to diagnose and mimic those of Chron’s disease. Total body computed tomography and laparotomy with an intestinal biopsy for the detection of *Mycobacterium tuberculosis* are the means of avoid the risks of a misdiagnosis in children with unexplained chronic abdominal problems.

## Background

Intestinal tuberculosis (ITB) and peritonitis are the two most common forms of abdominal tuberculosis [1]. In industrialised countries, where the risk of ingesting *Mycobacterium bovis* is now marginal because of the elimination of infected cattle and the availability of pasteurised milk, almost all cases of ITB are due to *Mycobacterium tuberculosis* (*Mt*) and secondary to the lymphohematogenous dissemination of bacteria from an infected site or the swallowing of sputum from an extensive pulmonary cavitation [1]. Relatively common in adults [2], ITB is considered rare in children and adolescents because epidemiological data collected several years ago indicate that only 1-5% of the pediatric cases of pulmonary tuberculosis (TB) are complicated by abdominal infection [3]. However, as many of the signs and symptoms of disease are mild in comparison with those due to extra-intestinal *Mt* infection [3], it is possible that the real incidence of ITB in children is higher and that a number of cases are not correctly diagnosed. A recent analysis of autopsy cases of children with TB found intestinal infection in 37.5%, most of which were diagnosed as having a pulmonary lesion, the primary cause of hospital admission and death [4].

The protean manifestations of ITB mean that the diagnosis is often delayed (sometimes even for years), thus leading to increased mortality and unnecessary surgery [3, 5]. The signs and symptoms frequently mimic those of many other conditions, such as acute abdominal carcinoma, perforation or appendicitis, but the main diagnostic dilemma is to differentiate ITB and Crohn’s disease (CD), particularly in children [1]. A misdiagnosis can have dramatic consequences: the initiation of immunosupressants or biological agents after a presumptive diagnosis of CD can lead to severe and sometimes fatal complications, such as the systemic dissemination of the infection and, in the case of a misdiagnosis of ITB, unnecessary anti-TB treatment can lead to a risk of toxicity and delay the treatment of CD.

Our case is a good example, and underlines the fact that diagnosing ITB is a challenge for pediatricians and requires a high index of suspicion.

## Case presentation

**Figure 1:**
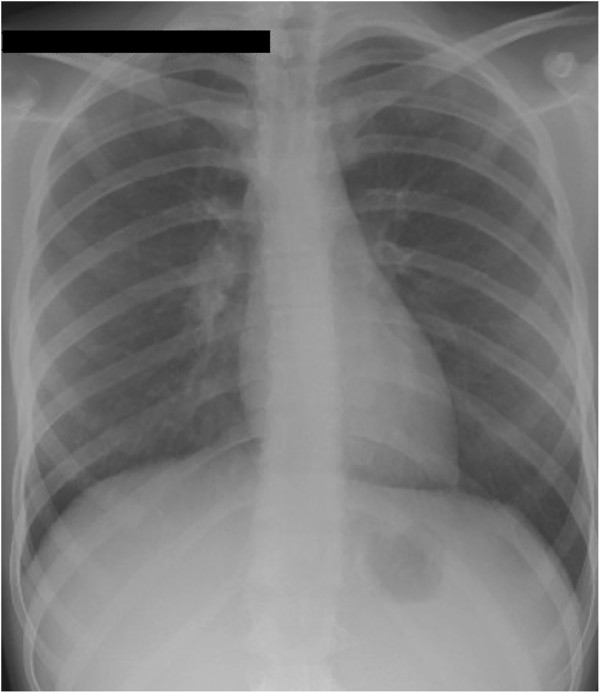
**A postero-anterior chest radiograph was normal.**

A 13-year-old Caucasian, Italian female adolescent attended our Emergency Department complaining of abdominal pain, a fever of up to 38°C, night sweats, diarrhea with blood in stool, and a weight loss of about three kilograms over the previous two months. Her family history was negative for any relevant diseases, particularly TB; furthermore, she, who was not BCG vaccinated, had not travelled abroad recently and no case of TB had been diagnosed among her school-fellows. Physical examination revealed a marked skin pallor and considerable abdominal distension with relevant discomfort in all the abdominal quadrant. No other abnormal signs and symptoms were found or reported. Laboratory tests revealed a decreased white blood cell count (WBC, 4,100/mm^3^) with anemia (Hb 7.6 g/dL), and increased C-reactive protein levels (1.18 mg/dL). The Mantoux tuberculin skin test (TST) was negative. A chest X-ray (Figure 1) and an abdominal ultrasonography did not reveal any significant findings. In particular, no acites was evidenced. After a blood transfusion, the patient underwent colonoscopy that showed diffuse mucosal congestion and significant blood loss, and laparatomy showed small bowel and colon loops with a whitish appearance. A biopsy of the ileal mucosa revealed inflammation with noncaseating granulomas possibly due to bacterial infection, and so only a standard bacterial culture was carried out. Given the suspicion of an opportunistic bacterial infection in a child with chronic inflammatory bowel disease (possibly CD), treatment with a third-generation cephalosporin was started (ceftriaxone 2 g/day).

**Figure 2:**
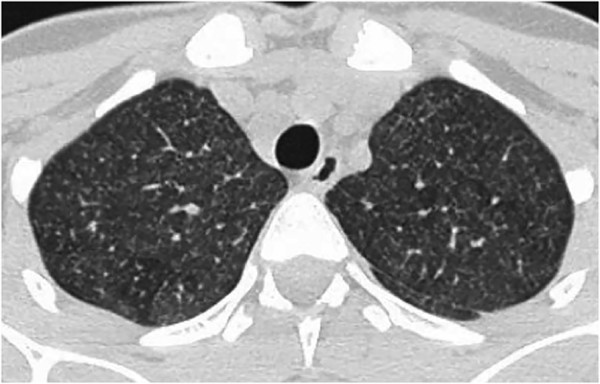
**Axial CT scan of the upper lobes of the lung showed numerous, bilateral, poorly defined and randomly distributed small pulmonary nodules with a preferentially centrilobular location.**

**Figure 3:**
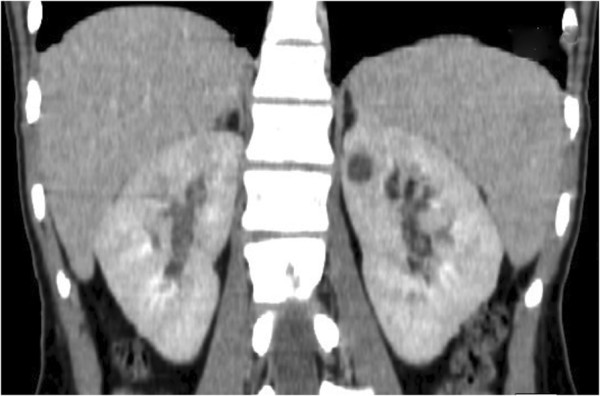
**Coronal multiplanar reconstruction (MPR) of the abdominal CT scan revealed a hypodense lesion in the upper pole of the left kidney.**

However, the abdominal pain, fever and poor general condition persisted and so, after 11 days, the patient underwent total body computed tomography (CT). Chest CT showed lower right lobe consolidation with coalescing nodules leading to patchy irregular opacities. The upper lobes contained numerous, bilateral, poorly defined and randomly distributed small pulmonary nodules with a preferentially centrilobular location (Figure 2). Other significant CT findings were a hypodense lesion in the left kidney (Figure 3) and lytic lesions in a number of vertebral bodies, without any demonstrable brain alterations. However, magnetic resonance imaging (MRI) of the brain revealed multiple nodular lesions in the subcortical white matter of both cerebellar hemispheres and medium-sized cerebellar peduncles; some of these lesions showed target signs of probable caseous necrosis.

On the basis of the radiological findings, miliary TB was suspected and bronchoscopy was performed together with a brochoalveolar lavage (BAL) in order to search for *Mt* by means of polymerase chain reaction (real-time PCR, Cobas TaqMan MTB PCR system) and culture. A urine sample was also tested for the same reason. Both cultures were positive, whereas PCR identified *Mt* only in the BAL fluid. Miliary TB was confirmed and treatment with four drugs was started in accordance with the WHO guidelines (isoniazid 10 mg/kg/day, rifampicin 15 mg/kg/day, ethambutol 20 mg/kg/day, and pyrazinamide 35 mg/kg/daily) [6]. The therapy was rapidly effective as the fever disappeared within a few days, the abdominal problems resolved, and the patient’s general condition improved together with a gain in body weight. Routine blood examinations were tested every week for the evaluation of drugs safety in the first month, and they were always in normal range. The patient was discharged one month after starting therapy with the indication to continue the four drugs for two months and to monitor blood examinations every two week. At the end of the second month of four-drug treatment, on the basis of the negative clinical condition it was decided to continue with isoniazid and rifampicin for further 7 months with blood examinations evaluated once every month. Total body CT and brain MRI six months after the beginning of anti-TB treatment confirmed the complete disappearance of all of the lesions in all body sites.

## Conclusions

This case study illustrates the importance of considering ITB in the differential diagnosis of all pediatric patients presenting with chronic, non-specific abdominal signs and symptoms even if the findings of an initial evaluation do not support diagnosis of TB. In our case, the diagnosis of ITB was not immediately considered because anamnestic data and TST were negative and no extra-intestinal localisation of TB was suspected at the time of the physical examination or chest radiography. Moreover, the clinical presentation, laboratory and abdominal ultrasonographyc data, and endoscopic and histological features were not significantly different from those usually found in patients with CD.

A negative personal and family history in a patient with TB is possible when (as in our case) the patient lives in a country with low incidence of *Mt* infection, the case is the first in the family, and the patient has had no demonstrable contact with potentially infected subjects. Any of the family members received anti-TB treatment and TST was negative in all of them. Furthermore, a negative TST is relatively common in patients with disseminated TB, which explains why T-cell based interferon gamma release assays (IGRAs) have increasingly replaced TST in the differential diagnosis of ITB and other intestinal disorders, particularly CD. A recent meta-analysis of the most important studies indicates that, although not sensitive enough, IGRAs are specific for ITB [7]. The false negative rate was only 13%, which is acceptable for clinical purposes and supports the systematic use of IGRAs when diagnosing chronic abdominal problems in children with a negative TST [7].

An apparently normal chest radiography is frequent in patients with ITB, and does not exclude a diagnosis. Radiographic features suggesting TB have been found in only 32% of patients with intestinal *Mt* infection [8]. As in our case, only CT can permit a complete evaluation of lung and extra-intestinal involvement. Like patients with CD, our patient only had constitutional symptoms (fever, anorexia and weight loss) associated with abdominal pain and distension.

Abnormalities in routine blood tests, such as total and differential leukocyte counts, an increased erythrocyte sedimentation rate, high C-reactive protein levels and low hemoglobin levels are also common to ITB and CD [9], and the laparoscopic and histological findings were similar to those that can be found in some cases of CD. In relation to biopsy results, it is usually considered that fissuring ulcers, lymphoid aggregates, trans-mural inflammation and non-caseating granulomas are fairly specific to CD, whereas large granulomas, often with caseation and confluence, suggest ITB [9]. However, the histological appearance of both ITB and CD can show non-specific changes, and even non-caseating granulomas can be seen in 35.9% of the biopsy specimens of ITB patients [9]. The differential diagnosis of such cases is therefore very difficult if only the histological features of biopsy specimens are considered and in cases with life-threatening condition an empiric treatment in case of suspected intestinal tuberculosis while waiting for biopsy results.

The most reliable differential method is to find evidence of *Mt* in the intestinal tissues. Unfortunately, acid fast bacilli staining lacks sensitivity and specificity; in addition, a biopsy culture for *Mt* is time-consuming (3–8 weeks) and the results are frequently negative. Identification of *Mt* by means of PCR can be easier and more effective. It has been reported that PCR positivity was 64.1% in 39 specimens from patients with intestinal tuberculosis, but zero in 30 specimens from patients with CD [10]. Moreover, 71.4% of tissue samples of intestinal tuberculosis with granulomas similar to those of CD (10/14) were positive by PCR, and 61.1% (11/18) were positive in ITB tissues without granulomas. Unfortunately, we did not use PCR on our biopsy specimen and the identification of ITB was only possible two weeks after hospitalisation, when total body CT revealed multiple lesions consistent with miliary TB, and PCR on BAL fluid identified *Mt*. On the other hand, also peritoneal TB represents a diagnostic challenge in children and adolescents and should be considered in patients with fever, abdominal pain, weight loss, and abnormal chest radiography; characteristic CT findings and a history of exposure to TB contribute to the diagnosis [11].

In conclusion, this case confirms that in children TB can be marginally symptomatic for a long period of time and that ITB can be very difficult to diagnose because its differentiation from CD can only be sure when extra-intestinal *Mt* infection is identified and the pathogen can be demonstrated by means of PCR in bioptic intestinal specimens. This means that total body CT eventually associated with laparotomy and intestinal biopsy for the detection of *Mt* are the only means of avoid the risks of a misdiagnosis in children with unexplained chronic abdominal problems.

## Consent

Written informed consent was obtained from the patient’s parents and a written informed assent was obtained from the patient for this study. A copy of the written informed consent and assent is available for this journal’s Editor-in-Chief to review.

## Abbreviations

BAL: Brochoalveolar lavage
CD: Crohn’s disease
CT: Computed tomography
IGRAs: Interferon gamma release assays
ITB: Intestinal tuberculosis
*Mt*: *Mycobacterium tuberculosis*
MRI: Magnetic resonance imaging
PCR: Polymerase chain reaction
TB: Tuberculosis
TST: Tuberculin skin test
WBC: White blood cell count.

## References

[1] Cruz AT, Starke JR (2007). Clinical manifestation of tuberculosis in children. Paediatr Resp Rev.

[2] Donoghue HD, Holton J (2009). Intestinal tuberculosis. Curr Opin Infect Dis.

[3] Al-Fadel SM, Al-Quorain A, Larbi E, al-Fawaz I, Taha O, Satti MB (1997). Tuberculous peritonitis in children: report of two cases and literature review. J Pediatr Gastroenterol Nutr.

[4] Ridaura-Sanz C, López-Corella E, Lopez-Ridaura R (2012). Intestinal/peritoneal tuberculosis in children: an analysis of autopsy cases. Tuberc Res Treat.

[5] Boukthir S, Mrad SM, Becher SB, Khaldi F, Barsaoui S (2004). Abdominal tuberculosis in children. Report of 10 cases. Acta Gastroenterol Belg.

[6] WHO: **RAPID ADVICE Treatment of tuberculosis in children.** Available at: http://whqlibdoc.who.int/publications/2010/9789241500449_eng.pdf?ua=1. Accessed on 18 March 2014 26269860

[7] Chen W, Fan JH, Luo W, Peng P, Su SB (2013). Effectiveness of interferon-gamma release assays for differentiating intestinal tuberculosis from Crohn’s disease: a meta-analysis. World J Gastrroenterol.

[8] Alvares JF, Devarbhavi H, Makhija P, Rao S, Kottoor R (2005). Clinical, colonoscopic, and histological profile of colonic tuberculosis in a tertiary hospital. Endoscopy.

[9] Almadi MA, Ghosh S, Aljebreen AM (2009). Differentiating intestinal tuberculosis from Crohn’s disease: a diagnostic challenge. Am J Gastroenterol.

[10] Gan HT, Chen YQ, Ouyang Q, Bu H, Yang XY (2002). Differentiation between intestinal tuberculosis and Crohn’s disease in endoscopic biopsy specimens by polymerase chain reaction. Am J Gastroenterol.

[11] Lin YS, Huang YC, Lin TY (2010). Abdominal tuberculosis in children: a diagnostic challenge. J Microbiol Immunol Infect.

